# Oral Health Epidemiological Investigation in an Urban Homeless Population

**DOI:** 10.3390/dj12100324

**Published:** 2024-10-08

**Authors:** Roberta Lione, Massimo Ralli, Francesca Chiara De Razza, Giuseppe D’Amato, Andrea Arcangeli, Luigi Carbone, Paola Cozza

**Affiliations:** 1 Department of Health Sciences, UniCamillus-Saint Camillus International Medical University, 00131 Rome, Italy; roberta.lione@unicamillus.org (R.L.); francescachiara.derazza@outlook.it (F.C.D.R.); giuseppe.damato@unicamillus.org (G.D.); 2 Department of Sense Organs, Sapienza University of Rome, 00186 Rome, Italy; 3 Department of Anaesthesiology and Intensive Care Medicine, Fondazione Policlinico Universitario A. Gemelli IRCCS, 00136 Rome, Italy; 4 Department of Emergency and Internal Medicine, Isola Tiberina Fatebenefratelli Hospital, Gemelli Isola, 00186 Rome, Italy

**Keywords:** dental care, health promotion, homeless, oral health, poverty, quality of life

## Abstract

The purpose of this clinical epidemiological investigation was to examine the oral health conditions of homeless people in the city of Rome, Italy. A total of 157 homeless subjects were subjected to a first dental visit, during which anamnestic information was recorded in a digital medical record. A diagnosis of dental disorders was performed by assessing oral hygiene conditions, periodontal health, the presence of caries and/or root residues, and the presence of partial and/or total edentulousness. Caries and missing teeth were evaluated by the DMFT index. The first major criticality was represented by poor or absent oral hygiene. The examined sample showed a major percentage of high DMFT (63.0%); the most common clinical condition was the presence of numerous root residues. Regarding periodontal health, 73.2% of patients had gingivitis, 21.6% periodontitis, while 11 patients had periodontal pathologies with tooth mobility (7%). Finally, 8.9% of patients had one or two missing elements, 22.9% had partial edentulousness, and 8.9% of the sample had total edentulism. This analysis provides an important basis for strengthening health promotion and the importance of accessible and effective care for this population. It will therefore be necessary to continue to adopt a patient-centered approach geared towards addressing the demands that this population faces in maintaining their oral health.

## 1. Introduction

Poor oral health is a common problem among vulnerable populations, such as people living in poverty and homelessness, who often experience higher levels of dental caries and periodontal diseases [1,2,3,4,5]. Furthermore, vulnerable groups are more likely to experience barriers to accessing dental services, leading to delayed or missed diagnoses and worse clinical presentation of dental diseases [3,4,6].

The incidence of homelessness is often variable and depends on several factors. The latest epidemiological survey conducted in Italy [7,8] showed that there were 96,197 homeless individuals in the country. Among them, 68% were men (65,407), while the remaining 32% (30,790) were women. Rome hosts the highest number of homeless people (over 22,000 people), followed by Milan (8541 people), Naples (6601) and Turin (4444). These data show that homeless people tend to be concentrated in large population centers, probably because there is a hope of more support and more structured care services [8,9]. Despite the high estimated numbers, it is difficult to make a precise assessment of this phenomenon: in fact, the numbers can vary seasonally, depending on social welfare policies and contingent economic conditions [9]. In addition, this vulnerable segment of the population can live inside shelters, temporary shelters, public places, and even on the streets, making it even more difficult to collect accurate data [10,11].

Homeless people in the city of Rome, Italy, represent a peculiar component of the population and their social, psychological, and physical well-being is always an important issue to be addressed. As a result, there is a need to meet their health needs [12,13].

Dental care is a fundamental aspect of an individual’s overall health; in Italy, it is characterized by a combination of public and private services, with a clear division between these sectors. Dental care is mostly accessed through the private sector, where patients are responsible for the full cost, or through private insurance. Access to such care for the homeless is often limited or non-existent, and significant difficulties are often faced in enabling these people to obtain adequate dental care free of charge. This reliance on private providers creates disparities in access to comprehensive dental care, particularly for lower-income populations, who may face financial barriers to obtaining necessary treatments [14].

The lack of free medical services and the introduction of free artificial intelligence technology led to the initiative of some patients to self-medication and self-diagnosis; however, these new technologies do not substitute the dental practice [15,16,17].

There is wide evidence that the absence of dental health and primary prevention can lead to serious overall health problems due to a lack of preventive care and timely treatments [1,14,18]. Homeless living conditions, including lack of access to adequate sanitation and a low-nutritional, high-sugar diet, contribute to worsening dental problems [18]. Furthermore, it is common to find in this population a lower awareness of individual oral health or the risks associated with lack of dental care, conditions that can lead to a worsening of the dental situation [19].

Last, the restrictive measures due to the recent Coronavirus Disease 2019 (COVID-19) pandemic have further limited the ability of homeless individuals to access dental care, causing restrictions, and increasing the risk of untreated dental issues, thus worsening overall health outcomes. This enhanced the existing disparities, as people experiencing homelessness already face substantial difficulties in accessing dental care [20,21,22,23,24].

Initiatives aimed at providing dental care to the homeless can make a difference in their quality of life and overall well-being. Non-governmental organizations and volunteers working with the homeless often point to the critical need for dental care within this population [15,25]. Vatican City offers free medical assistance to those living in poverty, marginalization, or difficulty. The Primary Care Services of the Dicastery for the Charity Services, located under the Colonnade of Bernini in St. Peter Square, provide healthcare services, including dental care, to homeless and vulnerable populations [26]. This clinical epidemiological investigation aims to report and discuss the oral health conditions of homeless people referring to this service, with a special focus on the treatment models adopted for this peculiar population.

## 2. Materials and Methods

This observational study was conducted following the Declaration of Helsinki and approved by the Institutional Ethics Committee of the Hospital of Rome “Tor Vergata” (no. 75.23, 1 September 2023). All patients provided were aware they were taking part in a project and signed a written informed consent before their enrolment.

The study included adult patients experiencing homelessness with at least one dental condition/poor oral health referred for dental evaluation to the Primary Care Services of the Dicastery for the Service of Charity from September 2023 to June 2024.

Patients were defined as “homeless” if they had been residing in a homeless shelter for a minimum of 7 consecutive days (sheltered homelessness) or indicated living on the street or in places not meant for human residency for at least 120 nonconsecutive days in the last 6 months (unsheltered homelessness) [27]. Participants were excluded if they were <18 years of age or did not match the criteria for homelessness.

### 2.1. Clinical Evaluation Protocol

Two trained dental examiners (PC, RL), with experience in dental pathology and general dentistry, carried out the first dental visit. A complete anamnestic evaluation was made using a digital medical record, filled in the presence of the patient. The patient’s name and surname, age, sex, country of origin, years spent in Italy, housing condition, work situation, tobacco and/or alcohol habits, systemic pathologies, and comorbidities were recorded. These details were self-reported by patients due to language barriers and the lack of medical records.

A clinical oral examination was then performed.

The clinical oral examination focused on periodontal health, the presence of caries and/or root residues, and the presence of partial and/or total edentulousness.

The oral hygiene conditions were assessed in the first dental visit, based on various criteria: plaque index, bleeding on probing (BOP), the number of lost teeth, and the presence of caries [28]. Absent oral hygiene was reported when there was a plaque index of 4 to 5, BOP on more than twenty dental sites, and an incidence of more than six dental caries and/or teeth loss; poor oral hygiene was detected when there was a plaque index of 2 to 3 and BOP on more than ten dental sites with an incidence of four to six dental caries and/or tooth loss; good oral hygiene if the plaque index was 0 or 2 in presence of maximum ten sites of BOP and one to three dental caries or dental loss. Caries and missing teeth were evaluated by the DMFT index (decayed, missing, filled teeth), according to the guidelines of the World Health Organization (WHO) [29].

Particular attention was paid to the analysis of conditions considered an emergency, such as clinical pictures of acute infection, functional limitations, and pain symptoms. Based on clinical examination, a diagnosis of dental disorder was recorded. At the end of the visit, according to the treatment needs of each patient, subsequent appointments were scheduled at three affiliated facilities: “Madonna della Fiducia” Clinic for oral surgery and dental hygiene; “Santa Marta” Dispensary for Conservative Dentistry and Oral Surgery; and “Vincentian Solidarity” Dental Center, for removable prostheses.

Data were exported from internal digital medical records into a Microsoft Excel database (Microsoft Corp., Redmond, DC, USA).

### 2.2. Statistical Analysis

The collected data were stored and analyzed using Prism Software version 9 (GraphPad Software LLC, Boston, MA, USA). A descriptive analysis was carried out for all the variables considered, to verify the presence of anomalous or missing data and to provide a concise overview of the analyzed sample. Subsequently, tables were constructed relating to the epidemiological indices used, and explanatory graphs were extrapolated.

## 3. Results

The study included 157 homeless subjects (124 males, 33 females), mean age of 50 years (range 27–83, standard deviation of 13 years), evaluated between September 2023 and June 2024. Forty-eight subjects (30.6%) were Italian, while the majority (69.4%, 109 patients) were non-Italian citizens. Most of the patients examined were from Eastern Europe (46 subjects, 42.2%), 34.9% from Africa (38 subjects), 14.7% from Southern Europe, excluding Italy (16 subjects), and 8.2% from South America (9 subjects).

Regarding behavioral habits, 42.2% (63 subjects) said they were smokers, 19.7% (31 subjects) said they only consumed alcohol, 29.9% admitted to using both substances (47 subjects), while the remaining 10.2% (16 subjects) reported not having addictions (Table 1).

The examined patients had several important systemic pathologies. The most frequent were hypertension (31.8%, 50 subjects), lung diseases (19.7%, 31 subjects), diabetes (12.7%, 20 subjects), thyroid diseases (5.7%, 10 subjects), various psychological disorders (5.1%, 8 subjects), osteoporosis (3.8%, 8 subjects), and HIV (1.9%, 3 subjects). 19.1% of the patients surveyed reported no systemic diseases or medication intake (Table 1).

The majority of patients examined were referred to the clinic for the presence of dental pain (52.2%, 82 subjects), 36.3% required an examination for edentulism (57 patients), and the remaining 11.4% (18 subjects) complained of bruxism and/or temporomandibular dysfunction.

On physical examination, the first major criticality was represented by a condition of poor or absent oral hygiene: 80 subjects (50.9%) had no oral hygiene, 53 subjects (33.7%) had poor oral hygiene, and only 15.3% had good oral hygiene (24 subjects).

As a direct consequence of severely poor oral hygiene conditions, 12.1% of patients (19 subjects) had caries affecting one or three dental elements, 3.8% (6 subjects) had caries affecting four or six dental elements, and 3.2% (5 subjects) had caries processes affecting more than six dental elements. The examined sample showed a major percentage of high DMFT (63.0%).

The lack of treatment of caries processes leads to the loss of tooth structure which leads to the most common clinical condition, represented by the presence of numerous root residues. Out of 157 patients, 9 subjects (5.7%) developed complications having at least one root residue, 17 subjects (10.8%) had more than four root residues, and 10 patients (6.4%) had more than six root residues. Root residues in the dental arch were often linked to infections in situ. Acute inflammation, such as dental abscesses, was observed in 10.8% of patients (17 subjects), while 4.5% had pulpitis, and another 4.5% had dental fractures (7 subjects each).

Regarding periodontal health, 73.2% of patients (115 subjects) had gingivitis, 21.6% periodontitis (34 patients), and 11 patients had periodontal pathologies with tooth mobility (7%). In addition, among the patients examined, 4.5% had unusual lesions of the oral cavity for which further investigation was necessary.

Finally, one of the primary reasons for seeking help was tooth loss. Among the patients, 8.9% (14 subjects) had one or two missing teeth, while 22.9% showed partial edentulousness (36 subjects). In total, 14 patients (8.9%) of the examined sample had total edentulism of one or both arches (Table 2).

**Figure 1:**
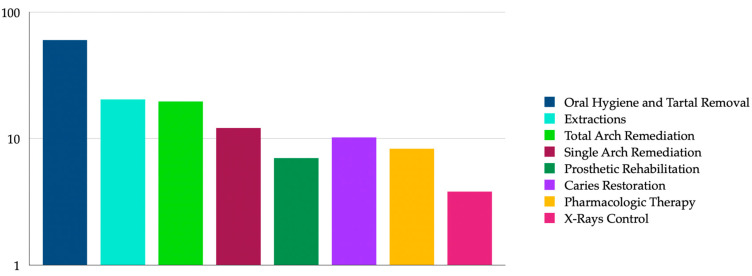
Graphical representation of treatment needs in the study population.

Regarding treatment requests, 94 patients (60%) required professional oral hygiene and tartar removal, 32 subjects (20.4%) required extractions, 31 patients (19.7%) required total remediation, 12.1% required rehabilitation prosthetics (19 patients), 10.2% of caries treatment (16 patients), 8.3% required painkiller and/or antibiotic therapy for pain treatment (13 patients), 11 patients (7%) needed remediation of a single arch, while 6 patients (3.8%) needed control X-rays (Figure 1).

Out of 157 initial consultations conducted at the Primary Care Services of the Dicastery, only 20 patients (13%) did not require any treatment. The remaining 137 patients (87%) were referred to specialized clinics to continue the dental care.

A total of 103 appointments were scheduled at the Clinic “Madonna della Fiducia” to carry out professional oral hygiene treatment (64.4%) and tooth extractions (36.6%). In addition, 22 appointments (14%) were scheduled at the “Vincentian Solidarity” Dental Center for prosthetic rehabilitation and 12 appointments (7.6%) at the “Santa Marta” Dispensary for conservative dentistry.

Given the complexity of their oral health conditions, 39 patients (24.8%) made more than one visit, obtaining different dental care in multiple appointments, for a total of 140 total dental visits from September 2023 to June 2024.

## 4. Discussion

The results of this study confirm a higher rate of dental conditions diagnosed at a more advanced stage among people experiencing homelessness compared to the general population, with specific characteristics. As widely described in homeless populations in different countries, the difficult accessibility of health services and the complex management of oral pathologies make the homeless population a particularly vulnerable part of society [1,2,15,30,31,32,33,34,35]. The results of our clinical epidemiological investigation confirm such difficulties and highlight important aspects of the general and oral health of our study sample.

The need for dental care for homeless people is a complex topic, requiring the attention and coordinated action of local authorities, non-governmental organizations, health professionals, and volunteers to ensure that all citizens, regardless of their social and economic status, have access to essential dental care [3,6,31]. A study from Bradley et al. evaluated the characteristics of available dental care models for people experiencing homelessness, indicating that services that are dedicated to treating these groups require flexible models of care in community-based settings to better manage specific population characteristics, such as sporadic patient attendance, high treatment needs, and complex care requirements [6].

It is important to note that the population under analysis in this investigation primarily consists of non-EU citizens, although a significant portion (30%) are Italians. The increase in foreign homelessness further increases the high level of vulnerability of this population. These data suggest that in addition to difficult bureaucratic administrative procedures, migrants face numerous practical obstacles, such as discrimination, and language and cultural barriers [27].

Most of the examined population sample reported smoking and consuming alcohol regularly, and they also declared drug consumption. Although the percentages regarding these habits may be underestimated, it is well recognized how these consumptions can lead to compromised oral health in the homeless population.

As far as dental pathologies are concerned, our data reveal a widespread presence of destructive caries, poor oral hygiene, and periodontal disease. These findings, which confirm data reported in other studies performed in comparable populations [17,36] represent the consequence of the difficulty in accessing preventive care and the lack of daily oral hygiene among the homeless population.

The eating habits of homeless people are highly dependent on the availability of food by the communities they serve. There is ample evidence that the diet of the patients under study is mainly based on easily usable foods and beverages with a high sugar content [37,38,39]. Therefore, a poor diet in combination with poor oral hygiene facilitates the formation of highly destructive carious processes [39]. In addition, smoking and alcohol abuse, behavioral habits present in most of the patients visited, worsen the health condition of the oral cavity, not to mention the systemic diseases commonly observed in the population (hypertension, diabetes, and lung diseases) [40]. Difficult pre-existing health conditions must be carefully managed during dental treatments to reduce the risk of complications [41].

Our study found that 87% of the examined sample presented with various oral diseases, indicating a need for dental care. Compared to previous studies from Italy [13,14,36], and considering the growing phenomenon of the homeless population, these results suggest that the rate of dental issues may increase significantly. For these individuals, dental care services often represent a significant financial burden, leading them to avoid dental visits, which results in unmet dental care needs. While homeless individuals living on the streets can access free care centers, the limited availability and accessibility of these medical centers create considerable challenges [42,43,44].

Treatment needs range from preventive procedures, such as professional oral hygiene, to more complex interventions, such as extractions of compromised elements and prosthetic rehabilitation procedures. It is therefore necessary to provide comprehensive and accessible dental services for this population, considering their specific needs and logistical limitations. Effectiveness in arranging appointments and ensuring patient participation is an important component, given the difficult lives of homeless people. Furthermore, it is essential to develop flexible follow-up strategies, to ensure that patients receive ongoing and substantial care [30].

Finally, the complexity of the cases treated is evidenced by the fact that many patients require more than one visit to complete the necessary treatments. This underlines the importance of a preventive as well as interventional approach to be able to intercept the most common oral pathologies in the homeless population from the outset, providing simpler and more timely treatment solutions [45].

### Limits of the Study

This study has some limitations. The small number of the sample examined, due to the limited duration of the observation period, the language barriers due to the large presence of non-Italian patients, and the difficulty of finding detailed information regarding the general health of the patients examined represent the major limitations of this study.

Another limitation of the study is the use of a convenience sample, involving only a selection of homeless participants available around Vatican City. Additionally, the description of this sample is limited to the observational period of the study and reflects only a small aspect of a larger phenomenon that requires more time to be fully studied and understood. However, the numbers presented are constantly updated due to the prospective nature of the project; therefore, they will be subsequently re-evaluated and updated to obtain a more comprehensive view of the phenomenon under consideration.

## 5. Conclusions

The analysis of epidemiological data and the need for dental treatment in the homeless population have highlighted the strong absence of suitable oral hygiene conditions and have framed a high need for different dental care. This study provides an important basis for strengthening health promotion and the importance of accessible and effective care for this population. It will therefore be necessary to continue to adopt a patient-centered approach, sensitive to the challenging social context and geared towards addressing the demands that this population faces in maintaining their oral health.

## Figures and Tables

**Table 1:** Descriptive epidemiology and comorbidities of the study sample.

	Patients (Number)	
Italian	48	30.6%
Non-Italian	109	69.4%
Non-Italian origins (n. 109)		
Eastern Europe	46	42.2%
Africa	38	34.9%
Southern Europe	16	14.7%
South America	9	8.2%
Behavioral habits		
Smoke consumption	63	40.1%
Alcohol consumption	31	19.7%
Both consumption	47	29.9%
None	16	10.2%
Comorbidities		
Hypertension	50	31.8%
Lung diseases	31	19.7%
Diabetes	20	12.7%
Thyroid diseases	9	5.7%
Psychological disorders	8	5.1%
Osteoporosis	6	3.8%
HIV	3	1.9%
None	30	19.1%

**Table 2:** Descriptive epidemiology of oral diseases.

	Patients (Number)	
Oral Hygiene		
Absent	80	50.9%
Poor	53	33.7%
Good	24	15.3%
DMFT		
Low	58	37%
High	99	63%
Decayed Teeth		
One or three dental elements	19	12.1%
Four or six dental elements	6	3.8%
More than six dental elements	5	3.2%
Missing Teeth		
One or more missing teeth	14	8.9%
Partial edentulism	36	22.9%
Total edentulism	14	8.9%
Root Residues		
One or three root remnants	9	5.7%
Four or six root remnants	17	10.8%
More than six root remnants	10	6.4%
Dental Abscesses	17	10.8%
Pulpits	7	4.5%
Dental Fractures	7	4.5%
Periodontal Health		
Gingivitis	115	73.2%
Periodontitis	34	21.6%
Tooth mobility	11	7%
Lesions of the Oral Cavity	7	4.5%

## Data Availability

All the data and materials used in this study are available upon reasonable request.
